# Are anxiety, depression, and stress distinguishable in Italian adolescents? an examination through the Depression Anxiety Stress Scales-21

**DOI:** 10.1371/journal.pone.0299229

**Published:** 2024-02-27

**Authors:** Sara Iannattone, Giuseppe Mignemi, Erika Pivetta, Michela Gatta, Claudio Sica, Valentina Cardi, Natale Canale, Andrea Spoto, Gioia Bottesi

**Affiliations:** 1 Department of General Psychology, University of Padova, Padova, Italy; 2 Department of Developmental and Social Psychology, University of Padova, Padova, Italy; 3 Woman and Child’s Health Department, Padova University Hospital, Padova, Italy; 4 Department of Health Sciences, University of Firenze, Firenze, Italy; National Cheng Kung University College of Medicine, TAIWAN

## Abstract

The adolescents’ ability to discriminate between different negative emotional states is still under debate. The Depression Anxiety Stress Scales-21 (DASS-21) serves as a useful tool to unravel this issue, yet the literature on its structural validity in young people is ambiguous. Therefore, this study aimed to expand knowledge on the emotional experience of youth by investigating the factor structure and psychometric properties of the DASS-21 in Italian adolescents. Six hundred fifty-five students (60.6% girls) aged 14–18 (*M* = 16.3 ± 1.29) completed an online survey containing the DASS-21 and Positive and Negative Affect Schedule (PANAS). To evaluate the factor structure of the DASS-21, several alternative models were tested, also adopting an Exploratory Structural Equation Modeling (ESEM) procedure. Measurement invariance, reliability, validity, and latent means differences were addressed. The ESEM model with three correlated factors of Depression, Anxiety, and Stress yielded the best fit to the data, supporting a hierarchical structure of the DASS-21. In addition, this model was invariant across sex and age groups. The Anxiety scale predicted both positive and negative affect, while Depression predicted positive affect only. Finally, girls scored higher than boys on Anxiety and Depression, but no age differences emerged. Overall, our results indicate that anxiety, depression, and stress are distinguishable in Italian adolescents but, simultaneously, share an underlying condition of general distress, which may explain the comorbidity between internalizing problems. Such findings are discussed in terms of clinical and preventive implications for the adolescent population.

## Introduction

Adolescence is a critical period characterized by high vulnerability to psychopathology [1]; as a matter of fact, the age range between 11 and 21 years represents the period where symptoms of most mental disorders emerge [2]. Moreover, according to the latest global data, updated to 2019, approximately 14% of the world’s adolescents have experienced at least one psychological disorder [3]. In particular, anxiety and depressive disorders (i.e., internalizing disorders) are among the most burdensome mental health problems affecting young people [4,5], with a prevalence rate ranging from 3.6% to 4.6% for the former and from 1.1% to 2.8% for the latter [3]. Importantly, these figures have likely increased in the wake of the COVID-19 pandemic, which have represented a significant source of psychological distress for children and adolescents worldwide [6]. Internalizing problems during developmental age are associated with several negative consequences [3] and often persist into adulthood [7], leading to severe impairment in numerous life domains (e.g., [8,9]). Of note, at a broadband level, internalizing disorders exhibit high homotypic stability (i.e., individuals are likely to remain in the same psychopathology domain over time); simultaneously, when focusing on specific manifestations within this category, heterotypic continuity is also frequently observed (e.g., transitions from anxiety symptoms to depressive symptoms and vice versa, or shifts from pure to comorbid disorders and vice versa) [10]. Therefore, early detection of these mental conditions is essential to prevent maladaptive outcomes and promote psychological well-being among youth.

Nevertheless, assessing and differentiating anxiety and depressive disorders can be particularly challenging for practitioners and researchers; indeed, the symptoms of these psychopathologies tend to overlap, as highlighted by their elevated comorbidity, especially in developmental age (e.g., [11]). A possible explanation for the frequent co-occurrence between anxiety and depression derives from the tripartite model proposed by Clark and Watson [12]. This model posits that the two internalizing subtypes share a nonspecific distress factor–namely, Negative Affectivity (NA)–which could partly account for the overlap between anxiety and depressive symptoms. In addition, the authors identified distinctive and unique components of anxiety and depression, stating that, although these conditions are both underpinned by NA, they are also independent of each other. Specifically, anxiety is distinguished from depression through the presence of Physiological Hyperarousal (PH; e.g., nervous tension, rapid breathing or heart rate), whereas depression is marked by low Positive Affect (PA; e.g., lack of energy, pleasure, interest, or enthusiasm) [12]. Although the tripartite model has been largely confirmed among adult samples (e.g., [13,14]), its applicability to youth populations is still under considerable debate; in fact, while some studies have provided empirical support to the tripartite model among both clinical (e.g., [15]) and nonclinical (e.g., [16–18]) adolescent samples, there is also evidence suggesting that anxiety and depression are indistinguishable in young people [19,20].

Bearing all this in mind, it seems clinically relevant to advance research on anxiety and depression in adolescence, also clarifying the extent to which these emotional states are experienced differently by adolescents. However, to promote progress in addressing this issue, valid and reliable instruments that evaluate internalizing distress in such a population are needed; in particular, self-report tools are considered extremely helpful because internalizing symptoms are personal and not always directly visible to others, especially in youth (e.g., [21]).

### The assessment of anxiety and depression through the Depression Anxiety Stress Scales

A valuable self-report questionnaire to assess internalizing symptomatology is the *Depression Anxiety Stress Scales* (DASS; [22]), a 42-item scale empirically developed to provide maximum differentiation between anxiety and depression in adults by reducing the importance of general symptoms and focusing on the distinctive symptoms of each construct. Although the DASS was originally designed to assess the core symptomatology of depression and anxiety, subsequent factor analysis studies pointed out a third factor including items that reflected nondiscriminating symptoms of the two syndromes and, in particular, some form of general tension; thus, the final version of the DASS resulted composed of three scales: (a) Depression, evaluating depressive symptoms such as hopelessness, dysphoria, low self-esteem, and anhedonia; (b) Anxiety, referring to subjective and physical symptoms of anxiety (e.g., breathing difficulties), and acute response to fear; (c) Stress, measuring tension, agitation, persistent arousal, and irritability [22]. Afterward, to facilitate the use of the DASS in time-constrained settings, a shorter 21-item version (i.e., DASS-21) was developed [23,24]. The DASS-21 has shown favorable psychometric and clinimetric properties; due to its short format, ease of administration, and availability in the public domain, it has become one of the most widely employed self-report instruments in research and clinical practice [25,26]. Notably, recent research has demonstrated its robust reliability and validity in specific populations, including primary and middle school teachers [27], undergraduate nursing students [28], frontline doctors [29], and caregivers of young children [30], thus substantiating its usability across various contexts. With specific regard to the factor structure of the DASS-21 in community adult samples, a bifactor model with a general factor (i.e., NA/general distress) and three domain-specific factors (i.e., Depression, Anxiety, Stress) is currently deemed the best solution [25,31], also in the Italian population [32]; in particular, such a solution has been considered consistent with the tripartite model [12] which hypothesizes the presence of a general factor comprising common symptoms of anxiety and depression as a possible reason for the high co-occurrence between these conditions [21].

#### The factor structure of the DASS-21 in adolescents

In contrast to substantial research addressing the structural validity of the DASS-21 in adult populations [25,31], a paucity of works have investigated its factor structure and psychometric properties in adolescent samples, also producing inconsistent findings. For example, the original DASS-21 model with three factors yielded the best fit in some studies carried out on different youth samples (i.e., Chinese primary [33] and middle school students [34], as well as adolescents aged 11–19 from Chile, Australia, China, and Malaysia [35]). However, all these studies revealed strong correlations between the Depression, Anxiety, and Stress factors, suggesting a potential lack of differentiation between these constructs. On the other hand, the original three-factor oblique solution failed to converge in two different empirical works conducted in Australia: Duffy et al. [19] proposed a two-factor solution of Physiological Arousal (composed of items assessing anxiety symptoms) and Generalized Negativity (including items assessing depression and stress symptoms) as the best-fitting model for adolescents aged 11–15 years, while Patrick et al. [20] provided support for a unidimensional factor structure in a sample of 11-17-year-old participants.

In light of the frequent conceptualization of anxiety and depression as potentially subsumed under a sort of higher-order framework [12,36], some studies have proposed a hierarchical model of the DASS-21 with a second-order factor of NA and two (Depression and Anxiety) or three (Depression, Anxiety, and Stress) first-order factors. Specifically, the former solution was supported in Confirmatory Factor Analysis (CFA) studies involving 12-18-year-old Australian [17] and Belgian [18] participants, while the latter better fitted the data from Vietnamese (Mean age = 16.5 ± 1.0 years; [37]), Malaysian (age range: 13–14 years; [38]), and Australian (age range: 11.83–15.57 years; [39]) adolescent samples. In addition, more recent research applying bifactor CFA found that a solution with a general NA factor and three domain-specific factors (Depression, Anxiety, Stress) was the best representation of the DASS-21 structure among Australian [21] and American [40] adolescents aged 12–18 and 14–17, respectively; however, in line with the results obtained in adult research [31], the general factor accounted for a high proportion of variance, while the three group factors lacked specificity, thus suggesting the use of a total score rather than separate scale scores.

Finally, Jovanović et al. [41] conducted a comprehensive analysis of the factor structure of the DASS-21 in a large sample of Serbian high school students (age range: 14–18 years). According to the authors, the main limitation of the models tested in previous works (i.e., multiple-correlated factors model and bifactor model) is that they do not consider cross-loadings of items, which instead can be expected in the DASS-21 due to the existence of partly overlapping components of anxiety, depression, and stress. Therefore, in an attempt to overcome such a shortcoming and fully explore the multidimensional factor structure of the DASS-21, they adopted an Exploratory Structural Equation Modeling (ESEM) framework [42]. This measurement method takes cross-loadings of all items on all factors into account, thus representing a promising alternative to evaluate the factor structure of the DASS-21. Furthermore, since ESEM does not consider the presence of a general factor within the scale, the authors also tested a bifactor-ESEM model [43], which assumes that items are associated not only with a general factor and their own specific factor but also with factors other than those they are designed to measure. The results showed that the bifactor-ESEM solution outperformed alternative models (i.e., the ESEM model and the factor structures tested in previous studies); more precisely, a strong general factor of psychological distress emerged and, simultaneously, the Depression and Anxiety (but not the Stress) subscales were found to possess a considerable amount of specificity over and above the general factor [41]. Therefore, the authors suggested that the total score and the Depression and Anxiety scale scores can be used with confidence, while calculating a separate score for the Stress subscale could be equivocal [41].

### The current study

The DASS-21 enables a comprehensive assessment of the symptoms of different negative affective states (i.e., anxiety, depression, and stress), providing an overview of internalizing experiences; therefore, it may represent a helpful tool to clarify whether adolescents are able or not to differentiate between different negative emotional states. Nevertheless, as previously mentioned, the literature on the factor structure of the DASS-21 in young people is still ambiguous. For instance, it seems unclear whether the use of separate scale scores assessing anxiety, depression, and stress is justified in adolescence or it would be more appropriate to calculate a total score of general distress. Furthermore, to date, no study has investigated whether the structure of the adult DASS-21 is generalizable to the Italian adolescent population; however, without evidence regarding the psychometric properties of the tool, the application of the adult scoring to youth can lead to erroneous conclusions. Based on these premises, the current study aimed to investigate the factor structure and psychometric properties of the DASS-21 in a group of adolescents (age range:14–18 years) from the general Italian population. By pursuing this objective, it is also possible to gain deeper insight into the structure of adolescents’ emotional experience as regards their ability to discriminate between anxiety, depression, and stress.

In the first place, following the recent evidence by Jovanović et al. [41], we might have expected the bifactor-ESEM model to best represent the factor structure of the DASS-21 among Italian adolescents. However, as outlined earlier, the literature is rather controversial, posing challenges in crafting a precise hypothesis. Thus, we systematically tested all models considered in previous studies (*see* Statistical analyses section for details); this approach was used not only to identify the optimal factorial solution, but also to ensure the comprehensiveness and comparability of our study within the existing literature. Second, we investigated Measurement Invariance (MI) across sex and age groups (14–16 years vs. 17–18 years); previous research generally found that the DASS-21 factor structure was invariant between boys and girls [18,37,38,41], while MI across age has been explored and supported only by a few studies [17,41]. Importantly, the decision to segment the age groups into 14–16 years and 17–18 years was mainly influenced by the seminal work of Jovanović et al. [41], who employed the same categorization. By adopting this approach, we aimed to facilitate a direct comparison between Jovanović et al.’s findings and our results, thereby excluding the possibility that divergent outcomes could be attributed to variations in age ranges. Then, complex latent mean differences across sex and age were addressed: Jovanović et al. [41] reported that girls scored higher than boys on anxiety and depression, while no age difference was shown; consequently, we hypothesized that similar results would emerge in the present study. Finally, we sought to examine convergent and divergent validity of the DASS-21: we investigated its associations with PA and NA and surmised that the specific factors (i.e., Depression, Anxiety, and Stress) were significant negative predictors of PA and positive predictors of NA. However, the only available study in this regard [41] found that only the Depression factor was a significant predictor of both PA and NA; therefore, this hypothesis was mainly exploratory.

## Materials and methods

### Participants

The sample included 655 White adolescents (394 girls, 60.6%) aged 14 to 18 (*M* = 16.3, *SD* = 1.29). 3.6% of participants attended the Italian third class of a lower secondary school (8^th^ grade), while the remaining percentage were recruited from upper secondary schools. Specifically, among the latter, 1.2% attended the first class (9 ^th^ grade), 5.6% the second class (10 ^th^ grade), 34.7% the third class (11 ^th^ grade), and 54.8% the fourth and fifth classes (12^th^ grade). Participants were asked if they had ever experienced psychological difficulties for which they sought professional help. Among those who responded (*n* = 566), 21.7% reported current or past psychological issues, such as anxiety-related difficulties, eating disorders, and family or school problems.

### Procedure

The data used in this work derive from three different but related studies, each employing an online survey with slightly different batteries of self-report questionnaires. The resulting independent datasets were merged to obtain the final sample of the present study. Consequently, not all students completed all the same self-report measures, but all completed the DASS-21. Each project was approved by the Ethics Committee for Psychological Research of the University of Padova (protocol number: 4332) and was carried out according to the recommendations of the Declaration of Helsinki.

Data were collected between 4^th^ October 2021 and 31^st^ May 2022 in lower and upper secondary schools located in Northern, Central, and Southern Italy. Following the approval of school directors, written informed consent was requested from parents of students younger than 18; for students aged 18, written consent was obtained directly. No incentives or rewards were given for participation.

Participants completed the online survey in a group setting in their schools during class time. Anonymity was ensured by using code numbers created by each student.

### Measures

The *Depression Anxiety Stress Scales-21* (DASS-21; Italian version by Bottesi et al. [32]) is a 21-item questionnaire measuring depression, anxiety, and stress symptoms, as well as general distress over the previous week on a four-point Likert scale (0 = *Did not apply to me at all*, 3 = *Applied to me very much*, *or most of the time*). The total score of the Italian version presented excellent reliability indices in both clinical (Cronbach’s α = 0.92) and nonclinical (Cronbach’s α = 0.90) adult samples.

The *Positive and Negative Affect Schedule–Trait version* (PANAS; Italian version by Terracciano et al. [44]) is a self-report measure that assesses two independent dimensions of PA and NA. The PA scale contains ten items referring to positive emotions (e.g., proud, determined, strong, excited, enthusiastic), while the NA scale is composed of ten items reflecting negative emotions (e.g., ashamed, hostile, irritable, upset, distressed). Participants rate the degree to which they usually experience each of the listed emotions on a five-point Likert scale, ranging from 1 = *Very slightly* to 5 = *Very much*. The Italian version of the tool showed adequate psychometric properties, with Cronbach’s α = 0.83 for PA and 0.87 for NA.

### Statistical analyses

The statistical validation of the scale was conducted through the following steps, with all analyses performed in MPlus, version 7.31 [45].

The first step focused on scale dimensionality, namely the detection of an optimal measurement model for the items. To this end, a CFA approach was adopted. However, in light of previous validation studies, a semi-confirmatory approach was also implemented within the ESEM framework. Following the seminal work of Jovanović et al. [41] and the literature on the DASS-21 factor structure in adolescent samples, the following nine models were tested:

*Model 1* –one-factor model with all items loading on a single factor [20];
*Model 2* –two-factor model with Depression scale items set to load on the first factor, and Anxiety and Stress scales items on the second factor [46];
*Model 3* –two-factor model comprising a PH factor (items 2, 4, 7 and 19) and a Generalized Negativity factor combining low PA and general NA items (remaining 17 items) [19];
*Model 4* –two correlated factors (Depression and Anxiety) with a second-order NA factor [17,18];
*Model 5* –three-factor model with three correlated factors of Depression, Anxiety, and Stress [33–35,37–39];
*Model 6* –three-factor model consistent with a tripartite model of anxiety and depression [12] with a PH factor (items 2, 4, 7 and 19), a low PA factor (items 3, 10, 16 and 21), and a general NA factor (the remaining 13 items);
*Model 7* –bifactor model with one general factor and three specific factors (Depression, Anxiety, and Stress [21,40]). This model allows each item to load both on the general factor and on a specific factor;
*Model 8* –ESEM model in which all items are specified to load on all three factors [41]. In line with previous applications of ESEM, an oblique geomin rotation was used relaxing the orthogonality assumption between the latent factors [47];
*Model 9* –bifactor-ESEM model in which all items were set to load on the general factor and cross-load on specific factors (oblique bi-geomin rotation was used) [41].

Considering the linearly ordered categorical response scale (i.e., four-point Likert scale) of the items, the robust weighted least squares (WLSMV) estimator was used. This is specifically designed for ordinal data and was shown to be robust to violations of normality; moreover, WLSMV was shown to provide less biased and more accurate estimates than robust maximum likelihood when adopted in ordinal data settings [48]. To evaluate model fit, the following indices were used: robust Satorra-Bentler scaled chi-square (SBχ^2^), Root Mean Square Error of Approximation (RMSEA), Standardized Root Mean Square Residual (SRMR), Comparative Fit Index (CFI), and Tucker–Lewis Index (TLI). Acceptable model fit was defined based on the recommended cut-off values: SBχ^2^ non-statistically significant; SBχ^2^ /*df* < 3; RMSEA less than 0.06, or between 0.06 and 0.08 for a reasonable fit; CFI and TLI greater than 0.95, or between 0.90 and 0.95 for a reasonable fit; SRMR less than 0.08 [49]. The SBχ^2^ value should be non-significant to indicate a good fit, but this is rarely obtained in large samples as the chi-square value is highly sensitive to sample size.

The second step was aimed at testing the invariance of the selected model and latent mean differences across sex and age groups. In this context, MI is meant as factorial invariance. When supported by evidence, the factor score of the respondent is independent of his or her own group membership and can be used for individual comparison purposes. A multiple-group semi-CFA framework was adopted to assess MI. The theta parametrization, in which residual variances for latent response variables are allowed to be parameters in the model but scale factors are not, was used in line with Jovanović et al. [41]. Different levels of MI exist and can be tested through several increasingly restrictive nested models. Due to the adopted semi-CFA approach, the following invariance steps were performed for both sex and age factors. The procedure started by specifying a configural invariance model in which equal factorial structures were imposed across groups. Following recommendations from the MPlus literature [50,51], uniqueness of each item was fixed to 1 in the first group for identifiability purposes. In the next step, scalar invariance was assessed by specifying a stricter model in which item intercepts were fixed equal across groups. In the Mplus program, metric MI is not allowed for ESEM using estimation for categorical data [45].

The last step aimed to verify convergent and divergent validity of the DASS-21. This was evaluated using the general SEM framework by examining associations with PA and NA as measured by the PANAS.

## Results

### Descriptive statistics

The mean scores obtained by participants on the DASS-21 Depression, Anxiety, and Stress scales were 8.29 (*SD* = 5.49), 7.11 (*SD* = 5.29), and 10.2 (*SD* = 5.06), respectively. These scores were calculated on the basis of the adult DASS-21 factor structure (32), namely: Depression: items 3, 5, 10, 13, 16, 17, 21; Anxiety: items: 2, 4, 7, 9, 15, 19, 20; Stress: items 1, 6, 8, 11, 12, 14, 18.

Descriptive statistics of empirical distribution of individuals’ responses (mean, standard deviation, skewness, and kurtosis) and inter-item Spearman’s correlations are shown in the (S1 and S2 Tables, respectively). There was a general trend of positive skewness and a slightly platykurtic shape, and the items were all significantly correlated, showing moderate to large effects.

### Factorial validity

The goodness-of-fit indices of the nine models are presented in Table 1. Models 1 and 3 exhibited the worst fit according to all indices. Models 2 and 6 showed a slightly better fit compared to the previous ones, though still falling short of acceptability. Models 4 and 5 demonstrated an acceptable fit to the data, even if the SBχ^2^ /*df* ratio was above the threshold of 3 for both models. Therefore, Models 1 to 6 were all excluded. Among Models 7 (bifactor CFA), 8 (ESEM), and 9 (bifactor-ESEM), all displayed optimal fit to the data with very similar indices, thus prompting a focused comparison. First, comparing Models 7 and 8, the former showed superior performance only in terms of SBχ^2^ /*df* ratio, while the latter demonstrated marginally better (Δ = |0.01|) SRMR and TLI values; the other fit indices remained consistent between the two models. Then, Model 9 (bifactor-ESEM) outperformed Model 7 in residual-based fit (indicated by a lower SBχ^2^ /*df* ratio) and showed a slight improvement (Δ = |0.01|) in TLI and SRMR values. However, compared with Model 8, Model 9 was less parsimonious (Model 8: 150 *df*; Model 9: 132 *df*) and introduced a marginal improvement in fit only in the SBχ^2^ /*df* ratio. No other differences in fit indices between Models 8 and 9 were observed.

**Table 1:** Fit indices of CFA and ESEM models.

Model	SBχ2 (*df*)	SBχ2 /*df*	RMSEA (90% CI)	SRMR	CFI	TLI
Model 1	1520.859 (189)	8.04	0.10 (0.99–0.10)	0.06	0.92	0.91
Model 2	958.761 (188)	5.09	0.08 (0.07–0.08)	0.05	0.95	0.95
Model 3	7533.023 (188)	40.06	0.24 (0.24–0.25)	0.15	0.58	0.53
Model 4	526.087 (174)	3.02	0.05 (0.05–0.06)	0.03	0.98	0.97
Model 5	679.350 (186)	3.65	0.06 (0.05–0.07)	0.04	0.97	0.97
Model 6	1186.585 (186)	6.37	0.09 (0.08–0.09)	0.05	0.94	0.93
Model 7	419.580 (165)	2.54	0.05 (0.04–0.05)	0.03	0.99	0.98
Model 8	414.521 (150)	2.76	0.05 (0.04–0.06)	0.02	0.99	0.99
Model 9	291.637 (132)	2.20	0.05 (0.04–0.06)	0.02	0.99	0.99

SBχ^2^ = Satorra-Bentler scaled chi-square; RMSEA = Root Mean Square Error of Approximation; SRMR = Standardized Root Mean Square Residual; CFI = Comparative Fit Index; TLI = Tucker–Lewis Index.

Given that Models 7, 8 and 9 demonstrated excellent and nearly identical fit indices, psychometric evidence alone seems insufficient to determine the best representation of the DASS-21 factor structure among these models. The literature underscores that the selection of the final model should be guided not only by psychometric performance but also by well-founded theoretical and interpretative reasons, especially when objective indices fail to provide a clear indication (e.g., [52]). Therefore, the following theoretical considerations come to the forefront. The introduction and application of the ESEM method for uncovering latent structure in the measurement of psychological constructs appears to be a flexible solution driven by realistic assumptions about item correlations. It is plausible, for instance, that items intended to measure a particular construct might be slightly but systematically correlated with different constructs, as in the case of the DASS-21. To be specific, from an interpretative standpoint, the DASS-21 ESEM model (Model 8) indicates that items are strongly influenced by target latent factors and minimally by non-target latent factors. In addition, the three latent factors are collectively influenced by a more general (i.e., second-order) factor, which is conceived as a broader construct encompassing Depression, Anxiety, and Stress. Thus, the ESEM model seems theoretically supported as a representation of the DASS-21 factor structure. On the contrary, in the DASS-21 context, a bifactor (Model 7) or bifactor-ESEM (Model 9) model could be particularly misleading. Indeed, CFA bifactor modeling imposes orthogonality between the general factor (i.e., general distress) and the specific factors (i.e., Depression, Anxiety, and Stress), thus assuming independence among the constructs; this assumption, however, does not seem realistic from a theoretical standpoint. Although in bifactor-ESEM modelling the orthogonality constraint is relaxed by specifying an oblique rotation among latent factors, even this solution does not seem an adequate representation of the DASS-21 structure. In fact, compared to the ESEM model (Model 8), bifactor-ESEM (Model 9) introduces an additional construct accounting for a common systematic variability among all 21 items beyond Depression, Anxiety, and Stress. Notably, the additional factor introduced in bifactor-ESEM is not a construct that includes the other three factors; instead, it assumes a source of variability that is common to all items but does not include the other three psychological constructs, thus potentially being “external” to them (e.g., common method bias).

For all the reasons outlined above, Model 8 should be preferred over Models 7 and 9 as it combines theoretical support, interpretability, and parsimony. A bifactor-ESEM model in this context could be deemed overly complex and lacking robust theoretical support, potentially resulting in an equivocal interpretation of what items are measuring. Therefore, prioritizing a clearer understanding becomes imperative, especially in the face of a non-decisive improvement in model fit.

All factor loadings of the ESEM model are reported in Table 2. The hierarchical structure of the scale (mathematically equivalent to a model with a second-level common factor) is suggested by the size and statistical significance of the respective factor loadings on the target factors. For the Depression factor, the target items displayed statistically significant loadings, with moderate to high correlations with this latent factor. A similar pattern was observed for the target items of both the Anxiety and Stress factors: their loadings were all statistically significant and demonstrated moderate to high effect sizes. Specifically, the target items of the Anxiety factor showed slightly higher factor loading values compared to those of the Depression and Stress factors. Furthermore, item loadings on the target factors were overall higher than those on non-target factors. The high omega coefficients for all the scales (*ω*s ranging from 0.81 to 0.93) and the total score (*ω* = 0.80) indicated very good to excellent score reliability estimates (Table 2).

**Table 2:** Standardized factor loadings and McDonald’s omega values of the ESEM solution.

Item	Depression	Anxiety	Stress
3 (Depression)	0.497***	-0.072	0.277
5 (Depression)	0.373***	-0.031	0.124
10 (Depression)	0.756***	0.023	0.027
13 (Depression)	0.594***	0.017	0.266
16 (Depression)	0.696***	-0.171	0.204**
17 (Depression)	0.712***	0.205*	0.017
21 (Depression)	0.872***	0.001	0.001
2 (Anxiety)	-0.113	0.536***	0.093
4 (Anxiety)	-0.157	0.861**	-0.278**
7 (Anxiety)	-0.211*	0.829***	-0.023
9 (Anxiety)	0.109	0.684***	-0.062
15 (Anxiety)	-0.033	0.808***	-0.243**
19 (Anxiety)	-0.185*	0.861***	0.020
20 (Anxiety)	0.009	0.678***	0.000
1 (Stress)	-0.097	0.366	0.344***
6 (Stress)	-0.002	0.344	0.667***
8 (Stress)	-0.042	0.194	0.489***
11 (Stress)	-0.010	-0.285	0.497***
12 (Stress)	0.224***	-0.311	0.459***
14 (Stress)	0.171*	0.220	0.479***
18 (Stress)	0.008	0.010	0.797***
*ω*	0.93	0.89	0.81

***p < 0.001,

**p < 0.01,

* *p* < 0.05.

*ω* = McDonald’s omega.

The standardized correlation coefficients between the first-level factors of the ESEM solution were all large in magnitude and significant (Depression-Anxiety: *r* = 0.712, Depression-Stress: *r* = 0.592, Anxiety-Stress: *r* = 0.785; all *ps* < 0.001).

Finally, descriptive indices (mean, standard deviation, percentiles) of the DASS-21 based on the ESEM solution are presented in the (S3 Table).

### Measurement invariance

Pertaining to sex, the configural model showed a good fit to the data, supporting the configural invariance of the DASS-21 across sex levels. Invariance with respect to the sex factor was also supported at the scalar level, showing a good fit and negligible changes in CFI and RMSEA (ΔCFI <0.001, ΔRMSEA <0.001).

Subsequently, MI across age was tested. The younger group was composed of adolescents aged 14–16 years (*n* = 349, 53.3%), whereas the older group included adolescents aged 17–18 years (*n* = 306, 46.7%), following the classification used by Jovanović et al. [41]. The configural model exhibited an adequate fit to the data. Scalar invariance was also supported, as shown by nonsensible changes in both CFI and RMSEA values between the configural and scalar models (ΔCFI <0.001, ΔRMSEA <0.001). Table 3 summarizes the abovementioned results regarding MI.

**Table 3:** Fit Indices and difference statistics for MI models (ESEM) across sex and age.

MI Model	SBχ2 (*df*)	RMSEA (90% CI)	Δ RMSEA	CFI	Δ CFI
*Sex*					
Configural	609.398 (321)	0.047 (0.044–0.049)	-	0.982	-
Scalar	664.607 (393)	0.046 (0.043–0.049)	<0.01	0.981	<0.01
*Age*					
Configural	565.806 (321)	0.048 (0.041–0.055)	-	0.986	-
Scalar	627.072 (393)	0.048 (0.042–0.055)	<0.001	0.997	<0.01

MI = Measurement Invariance; SBχ^2^ = Satorra-Bentler scaled chi-square; RMSEA = Root Mean Square Error of Approximation; CFI = Comparative Fit Index; Δ = Change.

### Latent means differences

Using the parameters of the ESEM model, the latent means across sex and age were compared. The latent means of boys and the younger adolescent group were fixed to zero, following the approach of Jovanović et al. [41]. The analyses of the latent means showed that girls reported higher levels of depression (unstandardized fitted mean(girls) = 1.011, SE = 0.120, *p* < 0.001) and anxiety (unstandardized fitted mean(girls) = 0.431, SE = 0.172, *p* < 0.05), but not higher levels of stress (unstandardized fitted mean(girls) = 0.281, SE = 0.138, *p* = 0.142). No significant age differences were observed in depression (unstandardized fitted mean(older) = −0.102, SE = 0.088, *p* = 0.181), anxiety (unstandardized fitted mean(older) = −0.145, SE = 0.081, *p* = 0.289), and stress (unstandardized fitted mean(older) = −0.077, SE = 0.086, *p* = 0.274).

### Convergent and divergent validity

The fit indices of the nine structural models of the DASS-21 were evaluated in the subsample of 312 participants who completed the PANAS. The associations between the DASS-21 and PANAS were then estimated. The ESEM model of the DASS-21 provided also in this subsample a good fit to the data (SBχ^2^ (134) = 294.710, *p* < 0.001, RMSEA = 0.04 (90% CI = 0.03, 0.05), CFI = 0.98, TLI = 0.97, SRMR = 0.03).

To assess both convergent and divergent validity of the DASS-21, a structural model was fitted, including direct paths from the ESEM solution to the PA and NA factors of the PANAS. Standardized path coefficients (Table 4) indicated that the Depression factor had a strong and negative association with PA but did not significantly predict NA. The Anxiety factor showed a positive association with NA and a negative one with PA, as expected. The Stress factor was not significantly associated with either PA or NA.

**Table 4:** Standardized path coefficients for the regression of PA and NA on the DASS-21 factors using the ESEM model.

DASS-21 factors	PA	NA
Depression	-0.422**	0.170
Anxiety	-0.429**	0.212*
Stress	0.053	0.153

**p < 0.001,

* *p* < 0.05.

DASS-21 = Depression, Anxiety, Stress Scales -21; PA = Positive Affect; NA = Negative Affect.

## Discussion

Internalizing problems in adolescence are extremely common and cause a considerable burden of disease, as they are associated with numerous short- and long-term negative consequences, such as suicide, alcohol and substance use, social withdrawal, poor school performance, and difficulties with peer relationships [3]. Despite their widespread impact, research on this topic is still plagued by unsolved issues, among which one of the most important concerns the high comorbidity between internalizing problems; specifically, it remains uncertain whether young people can effectively distinguish between different negative emotional states. The present study attempted to overcome such a limitation by exploring the factor structure and psychometric properties of one of the key tools for assessing internalizing symptoms–namely, the DASS-21 –in adolescents from the general Italian population. The study’s principal findings partially align with the hypotheses and offer novel insights into the structure of adolescent emotional experience as follows.

Among the several alternative factor models tested, only Models 7 (i.e., bifactor model), 8 (i.e., ESEM model), and 9 (i.e., bifactor-ESEM model) reached acceptable values for all considered fit indices. Specifically, in line with the results of Jovanović et al. [41], the bifactor-ESEM model outperformed competing models, albeit with only marginal fit improvement compared to the bifactor and ESEM models. Indeed, the fit indices of Models 7 to 9 were roughly the same, providing limited psychometric evidence to favor one model over the others. Therefore, the following theoretical considerations were drawn. The limitations of the bifactor model and bifactor-ESEM model (i.e., overcomplexity and only partial coherence with the DASS-21 theoretical framework) risked making interpretation difficult and leading to erroneous conclusions. Conversely, the ESEM model not only exhibited almost identical fit indices to the other models but also showcased its strengths by being more parsimonious than the bifactor-ESEM model; furthermore, it offered the notable advantage of enabling a clearer and theoretically grounded interpretation of the factor structure of the questionnaire (*see* the Results section for a more in-depth explanation). Consequently, the ESEM model was finally deemed the best representation of the DASS-21 structure among Italian adolescents. In this solution, all items significantly loaded on their specific target factors, with moderate to elevated values (all > 0.30); in particular, item loadings on each factor were generally higher than those found in the Italian adult population [32] and in most adolescent studies that supported a tripartite structure of the DASS-21 using bifactor-ESEM [41], traditional CFA [37,39], and bifactor CFA [21] approaches. Some considerations can be made with respect to items #1 and #5, which had notably lower loadings on their target factors (i.e., Stress and Depression, respectively) compared to the other items. To be specific, the result regarding item #1 (“I found it hard to wind down”) could be understood in light of Szabó [39] and Le et al. [37]’s observations, according to which some of the DASS-21 items assessing tension/stress among adults may not fully capture this construct among adolescents. In particular, it could be that the difficulty of winding down is not strongly representative of a state of stress in Italian adolescents, although further investigations are needed to corroborate this speculation. Additionally, the lowest loading of item #5 (“I found it difficult to work up the initiative to do things”) on its factor echoes previous findings on adolescent samples [17,18,21,35,37,39], suggesting that difficulty taking the initiative to do things is not specifically indicative of a depressive state in adolescence. This result also resonates with what was observed in the Italian validation study [32]; consequently, linguistic and cultural aspects can add to age-related factors in explaining the performance of this item. Further research involving different cultural and age groups may be helpful in unravelling this issue [53].

Some Anxiety, Depression, and Stress items were also found to load significantly on non-target factors, pinpointing the presence of partly overlapping components of anxiety, depression, and stress and thus the theoretical relevance of adopting an ESEM framework when studying these constructs. However, consistently with Jovanović et al. [41], items loaded higher on their target factor than on non-target factors, highlighting that the Anxiety, Depression, and Stress scales also have some degree of specificity. In addition, the elevated ω values point out that all the scales have excellent reliability.

Subsequently, the three factors emerged to be highly and significantly associated, in keeping with the strict relation and frequent comorbidity between anxiety, depression, and stress in adolescent age (e.g., [11]). Nevertheless, despite the large magnitude of the correlation coefficients between these dimensions, they were comparatively lower than those reported in most adolescent studies [17,18,33,34,38–40]. Specifically, the correlation coefficients between Anxiety and Depression as well as between Depression and Stress would be indicative of considerable distinctness between these constructs; on the contrary, those between Anxiety and Stress seem to indicate empirical overlap [54]. This last result raises doubts about the distinguishability between anxiety and stress in Italian adolescents. However, it should be kept in mind that anxiety often involves irritability and high arousal [55], which are aspects measured by the Stress scale as well; therefore, this could play a role in the adolescents’ difficulty in fully distinguishing between anxiety and stress.

Our study also accrued evidence to support the ESEM model’s invariance in different age and sex groups, in line with previous research [17,18,37,38,41]. This is an extremely relevant result, as it ensures that mean differences in the DASS-21 scale scores are unbiased for younger and older adolescents, as well as for girls and boys; in other words, mean differences truly reflect variability in anxiety, depression, and stress levels between groups. As a consequence, in the Italian context, the DASS-21 Anxiety, Depression, and Stress scores can be used to make inferences across sexes and throughout the adolescent lifespan. Future studies should consider expanding the age range by including 10-13-year-old adolescents as well, thus covering the whole adolescent age and providing an exhaustive overview of this issue.

Important clinical-theoretical and practical considerations on internalizing distress in adolescence can be drawn from the above findings.

First, the distinguishability between anxiety, depression, and stress among adolescents has been questioned by some studies [19,20,33]. Nonetheless, our results suggest that Italian adolescents are able to differentiate between unpleasant affective states, at least with regard to anxiety and depression, and depression and stress. This differentiation ability may be attributed to some phase-specific characteristics of adolescent development, such as improvement in deductive reasoning and self-reflective thinking [56]. Simultaneously, emotional and cognitive development is still maturing during this phase, and this can contribute to hindering a complete distinction between the anxiety and stress dimensions (at least as assessed by the DASS-21) [39]. Additional studies are needed to develop a more comprehensive understanding of these dynamics.

Furthermore, the observation that anxiety and depression, as well as depression and stress are highly associated but not overlapping constructs could mean that Italian adolescents conceptualize and experience these affects as distinct of each other. At the same time, the ESEM model supported a hierarchical factor structure of the DASS-21, thus pointing to the presence of a broader factor (i.e., general distress) encompassing depression, anxiety, and stress elements. Consequently, it would seem that anxiety, depression, and stress, while representing separate syndromes, also converge in the “general distress” trait in adolescence. Taken together, current data are consistent with the tripartite model proposed by Clark and Watson [12]. Indeed, they provide support to the presence of both common and distinctive elements of anxiety and depression (and stress), as well as to the existence of a general distress factor underpinning these negative emotional states and thus possibly accounting for their co-occurrence. In practical terms, this would justify the use of both separate scale scores and a total score of the DASS-21 in Italian adolescents.

The above-outlined issues can be further elucidated by taking into consideration the results on convergent and divergent validity. The general pattern of associations diverged somewhat from the results by Jovanović et al. [41]. Indeed, the Anxiety factor was found to significantly predict both PA and NA (despite being more closely associated with PA), while the Depression factor emerged to be a significant predictor of PA only. Instead, in line with Jovanović et al. [41], the Stress factor was not related to either PA or NA. First, the findings on anxiety and depression would suggest that, in the Italian adolescent population, low PA is common to both affective states, while high NA is specific to anxiety. Low PA may thereby be another factor besides general distress explaining the comorbidity between anxiety and depressive symptoms in adolescent age. Indeed, as Watson [57] argued, “more than one nonspecific factor is required to model comorbidity adequately” (p.19). On the one hand, this evidence is in contrast with the tripartite model [12], which posits that low PA is specific to depression; on the other hand, it is in line with such a model (and with the other findings of the present study) insofar as it further supports the notion that anxiety and depression share common characteristics but also exhibit peculiar elements making them distinguishable. The contrasting results between the present study and Jovanović et al. [41] call for further cross-cultural research delving into the relation between NA, PA, anxiety, and depression in the adolescent population. Subsequently, pertaining to the Stress factor, it emerged to be separate from both NA and PA. This finding aligns with the original conceptualization of stress by Lovibond and Lovibond [24] who stated that this construct is independent of NA. Furthermore, it can help shed some light on the raised question of the distinguishability between anxiety and stress in Italian adolescents; in fact, it shows that, although strongly associated, these two conditions do not completely overlap since they are not characterized by the same elements. Thus, these data challenge previous findings questioning the use of the DASS-21 Stress scale as a measure of a distinct affective state in adolescents [17,33,34,37,39].

Finally, we investigated latent means differences in the DASS-21 Anxiety, Depression, and Stress scale scores between girls and boys and younger and older adolescents. Consistently with the results by Jovanović et al. [41] and our hypotheses, the findings revealed sex differences in anxiety and depression, while no age differences emerged. To be specific, girls reported greater levels of depression and anxiety than boys. This result is in line with the commonly observed higher prevalence of internalizing symptoms in girls (especially during the adolescent period) and may be explained by some dispositional characteristics, such as girls’ heightened reactivity and ruminative tendencies (e.g., [58,59]). On the other hand, the absence of age differences could indicate that, in Italian adolescents, the developmental trajectories of anxiety, depression, and stress do not undergo significant changes with age, displaying a relatively stable trend. However, it should be also borne in mind that results could vary by considering different age groups, as major and rapid emotional changes occur during adolescence [60], particularly as regards the different facets of negative affect [61]. More data, preferably longitudinal, are required to shed light on the pathways of unpleasant affective states during this developmental phase.

Despite the intriguing results, a number of limitations need to be considered. First, the sample was composed of high-school students from a single cultural context, thus limiting the generalizability of the findings to different populations. Since the ESEM framework seems a valuable way to fully capture the multidimensional structure of the DASS-21, it is crucial for future investigations to test whether this approach remains effective across different cultures. Importantly, it should be noted that current literature supports modelling the DASS-21 factor structure using a bifactor approach [21,31,40,41]. However, a general better fit of bifactor models, as well as the slight fit improvement provided by the bifactor-ESEM model in the present study, could be due to the fact that these models are able to better accommodate implausible response patterns, thus tending to overfit data; for this reason, caution is usually recommended against interpreting bifactor models as substantive models [62]. Future work should consider resume the long-standing issue of the validity of bifactor models as representing the factor structure of the DASS-21. Another shortcoming of the present study is that it was conducted on adolescents from the general population only; therefore, subsequent research should also involve adolescents diagnosed with anxiety and depressive disorders to test the known-group validity of the questionnaire. Additionally, it is noteworthy that 21.7% of participants disclosed past or current psychological difficulties. In contextualizing this figure, it should be considered that the inherent psychological challenges faced by adolescents make them particularly prone to experiencing psychological difficulties; as a result, the inclusion of a modest proportion of participants reporting these issues is customary in research on adolescent samples. In this study, the above prevalence encompasses individuals with *general* psychological difficulties, which may not necessarily translate to diagnosed psychopathologies. Hence, this inclusion reflects the nuanced nature of our sample and provides a more ecologically valid representation of the general Italian adolescent population. However, it cannot be ruled out that the presence of psychological problems in a limited percentage of participants may have introduced bias to the results, potentially contributing to higher observed mean scores. Lastly, due to the cross-sectional design, we could not test the longitudinal MI of the DASS-21 structure, which should be examined in future studies to provide a more comprehensive understanding of the stability of the questionnaire’s factor structure over time.

## Conclusions and practical implications

In conclusion, our study provided support to the tripartite structure of the DASS-21 and showed that it is a valid and reliable questionnaire to evaluate internalizing symptomatology in Italian adolescents. In particular, from a practical standpoint, separate scale scores as well as a total score can be used with confidence to assess anxiety, depression, stress, and general distress in this population. The possibility to reliably assess and differentiate between unpleasant emotional states is crucial for research aiming to pinpoint the common and specific etiological factors and proximal mechanisms underlying each state.

From a clinical perspective, a tripartite structure of negative affective states emerged as a robust theoretical model for adolescents. This represents a crucial finding, since understanding the structure of youth’s emotional experience has a bearing on the treatment and prevention of internalizing disorders in this vulnerable population. Particularly, our findings indicate that anxiety, depression, and stress are distinct syndromes in Italian adolescents, yet they share an underlying condition of general distress; hence, this factor may contribute to explaining the comorbidity between internalizing disorders not only in adulthood, but also in adolescence. Moreover, low PA may add to general distress in specifically accounting for the co-occurrence between anxiety and depressive symptoms in adolescents. Conversely, high NA appears to be an exclusive component of anxiety, thus being particularly relevant to consider in the differential diagnosis with depression. These latter aspects, somewhat in contrast with the adult tripartite model [12], highlight that anxiety and depression may also have age-typical characteristics which should be taken into account in the nosology and assessment of these syndromes.

As noticed above, anxiety, depression, and stress resulted to be marked by both specific and nonspecific elements, all of which need to be considered to gain a whole and accurate picture of internalizing distress in the adolescent population [12]. In terms of clinical practice, the presence of common causal factors for depression, anxiety, and stress justifies the implementation of transdiagnostic interventions, which are fundamental in light of the frequent co-occurrence between internalizing problems. In particular, in case of adolescents with comorbid anxiety and depressive disorders, clinicians should consider targeting the nonspecific general distress and low PA symptoms to treat both disorders simultaneously. This approach aims to reduce the risk of relapse and enhance overall treatment effectiveness. Moreover, interventions designed towards such transdiagnostic symptoms may also be useful in case of isolated internalizing problems, potentially preventing the onset of future psychological disorders; indeed, consistently with the notion of heterotypic continuity, a symptomatic expression may beget an alternative symptomatic expression over the course of development (e.g., [63,64]). This is particularly pertinent for internalizing problems in adolescent age, as the manifestations within this broad category often display high heterotypic continuity patterns; for example, it has been found that anxiety symptoms often transform into depressive symptoms, and the presence of a major depressive episode is as a strong predictor of a subsequent anxiety disorder [65]. Finally, knowing the differences between internalizing subtypes is also meaningful, as it enables the development of early, syndrome-specific screening and prevention programs that can avoid progression to more severe symptomatology and promote psychological well-being; hence the importance of a questionnaire able to accurately measure the symptoms of each syndrome separately.

## Supporting information

S1 Table Descriptive statistics of items’ response categories of the DASS-21. (DOCX)

S2 Table Spearman’s *rho* correlations between the items of the DASS-21. (DOCX)

S3 Table Descriptive indices of the DASS-21 in the Italian adolescent population (*N* = 655). (DOCX)
