# Supplementary material for: Are anxiety, depression, and stress distinguishable in Italian adolescents? an examination through the Depression Anxiety Stress Scales-21
Source: PLoS One. 2024 Feb 27;19(2):e0299229. doi: 10.1371/journal.pone.0299229 (PMC10898757; doi:10.1371/journal.pone.0299229)
Supplement: S1 Table — (DOCX) [file pone.0299229.s001.docx]

**Are anxiety, depression, and stress distinguishable in Italian adolescents? An examination through the Depression Anxiety Stress Scales-21**

**Supporting information 1**

**S1 Table. Descriptive Statistics of Items’ Response Categories of the DASS-21**

| Item | Mean (*SD*) | Skewness | Kurtosis |
| --- | --- | --- | --- |
| 3 (Depression) | 1.17 (0.91) | 0.44 | -0.59 |
| 5 (Depression) | 1.43 (0.96) | 0.20 | -0.93 |
| 10 (Depression) | 0.98 (1.01) | 0.74 | -0.58 |
| 13 (Depression) | 1.39 (0.98) | 0.16 | -0.98 |
| 16 (Depression) | 1.03 (0.96) | 0.57 | -0.66 |
| 17 (Depression) | 1.33 (1.04) | 0.21 | -1.26 |
| 21 (Depression) | 0.93 (1.06) | 0.79 | -0.69 |
| 2 (Anxiety) | 1.10 (0.97) | 0.47 | -0.80 |
| 4 (Anxiety) | 0.87 (0.86) | 0.79 | -0.47 |
| 7 (Anxiety) | 0.98 (1.01) | 0.67 | -0.73 |
| 9 (Anxiety) | 1.28 (1.00) | 0.28 | -1.00 |
| 15 (Anxiety) | 0.88 (1.03) | 0.85 | -0.55 |
| 19 (Anxiety) | 1.03 (1.01) | 0.56 | -0.84 |
| 20 (Anxiety) | 0.94 (0.97) | 0.72 | -0.54 |
| 1 (Stress) | 1.22 (0.92) | 0.37 | -0.68 |
| 6 (Stress) | 1.35 (0.97) | 0.22 | -0.93 |
| 8 (Stress) | 1.43 (0.95) | 0.10 | -0.98 |
| 11 (Stress) | 1.97 (0.91) | -0.38 | -0.90 |
| 12 (Stress) | 1.49 (0.98) | 0.04 | -1.01 |
| 14 (Stress) | 1.19 (0.94) | 0.36 | -0.77 |
| 18 (Stress) | 1.50 (0.98) | 0.07 | -1.01 |
