# Supplementary material for: Are anxiety, depression, and stress distinguishable in Italian adolescents? an examination through the Depression Anxiety Stress Scales-21
Source: PLoS One. 2024 Feb 27;19(2):e0299229. doi: 10.1371/journal.pone.0299229 (PMC10898757; doi:10.1371/journal.pone.0299229)
Supplement: S2 Table — (DOCX) [file pone.0299229.s002.docx]

**Are anxiety, depression, and stress distinguishable in Italian adolescents? An examination through the Depression Anxiety Stress Scales-21**

**Supporting information 2**

| **Item** | **1** | **2** | **3** | **4** | **5** | **6** | **7** | **8** | **9** | **10** | **11** | **12** | **13** | **14** | **15** | **16** | **17** | **18** | **19** | **20** | **21** |
| --- | --- | --- | --- | --- | --- | --- | --- | --- | --- | --- | --- | --- | --- | --- | --- | --- | --- | --- | --- | --- | --- |
| 1 | 1 |  |  |  |  |  |  |  |  |  |  |  |  |  |  |  |  |  |  |  |  |
| 2 | 0.366 | 1 |  |  |  |  |  |  |  |  |  |  |  |  |  |  |  |  |  |  |  |
| 3 | 0.522 | 0.314 | 1 |  |  |  |  |  |  |  |  |  |  |  |  |  |  |  |  |  |  |
| 4 | 0.495 | 0.375 | 0.430 | 1 |  |  |  |  |  |  |  |  |  |  |  |  |  |  |  |  |  |
| 5 | 0.397 | 0.339 | 0.446 | 0.321 | 1 |  |  |  |  |  |  |  |  |  |  |  |  |  |  |  |  |
| 6 | 0.524 | 0.282 | 0.414 | 0.338 | 0.357 | 1 |  |  |  |  |  |  |  |  |  |  |  |  |  |  |  |
| 7 | 0.452 | 0.398 | 0.406 | 0.570 | 0.321 | 0.364 | 1 |  |  |  |  |  |  |  |  |  |  |  |  |  |  |
| 8 | 0.573 | 0.358 | 0.525 | 0.418 | 0.422 | 0.494 | 0.505 | 1 |  |  |  |  |  |  |  |  |  |  |  |  |  |
| 9 | 0.501 | 0.369 | 0.465 | 0.464 | 0.396 | 0.394 | 0.467 | 0.461 | 1 |  |  |  |  |  |  |  |  |  |  |  |  |
| 10 | 0.403 | 0.228 | 0.486 | 0.323 | 0.443 | 0.292 | 0.300 | 0.405 | 0.418 | 1 |  |  |  |  |  |  |  |  |  |  |  |
| 11 | 0.530 | 0.369 | 0.551 | 0.442 | 0.453 | 0.412 | 0.377 | 0.565 | 0.459 | 0.411 | 1 |  |  |  |  |  |  |  |  |  |  |
| 12 | 0.574 | 0.327 | 0.565 | 0.434 | 0.441 | 0.380 | 0.378 | 0.550 | 0.448 | 0.434 | 0.652 | 1 |  |  |  |  |  |  |  |  |  |
| 13 | 0.513 | 0.314 | 0.630 | 0.441 | 0.455 | 0.411 | 0.412 | 0.514 | 0.521 | 0.543 | 0.570 | 0.576 | 1 |  |  |  |  |  |  |  |  |
| 14 | 0.433 | 0.270 | 0.452 | 0.317 | 0.429 | 0.457 | 0.292 | 0.425 | 0.423 | 0.343 | 0.376 | 0.414 | 0.428 | 1 |  |  |  |  |  |  |  |
| 15 | 0.568 | 0.302 | 0.500 | 0.624 | 0.319 | 0.377 | 0.542 | 0.475 | 0.573 | 0.402 | 0.481 | 0.507 | 0.538 | 0.375 | 1 |  |  |  |  |  |  |
| 16 | 0.423 | 0.269 | 0.606 | 0.381 | 0.477 | 0.348 | 0.315 | 0.428 | 0.420 | 0.504 | 0.451 | 0.494 | 0.588 | 0.390 | 0.443 | 1 |  |  |  |  |  |
| 17 | 0.410 | 0.290 | 0.556 | 0.385 | 0.404 | 0.349 | 0.382 | 0.412 | 0.481 | 0.580 | 0.434 | 0.479 | 0.636 | 0.356 | 0.506 | 0.545 | 1 |  |  |  |  |
| 18 | 0.489 | 0.272 | 0.475 | 0.339 | 0.372 | 0.546 | 0.390 | 0.565 | 0.389 | 0.346 | 0.522 | 0.501 | 0.448 | 0.470 | 0.389 | 0.396 | 0.380 | 1 |  |  |  |
| 19 | 0.510 | 0.398 | 0.399 | 0.524 | 0.307 | 0.323 | 0.542 | 0.489 | 0.454 | 0.332 | 0.413 | 0.414 | 0.419 | 0.364 | 0.541 | 0.335 | 0.389 | 0.365 | 1 |  |  |
| 20 | 0.475 | 0.380 | 0.459 | 0.465 | 0.365 | 0.404 | 0.476 | 0.474 | 0.571 | 0.392 | 0.468 | 0.478 | 0.498 | 0.393 | 0.547 | 0.396 | 0.438 | 0.410 | 0.507 | 1 |  |
| 21 | 0.382 | 0.239 | 0.532 | 0.331 | 0.427 | 0.335 | 0.343 | 0.421 | 0.407 | 0.603 | 0.421 | 0.446 | 0.610 | 0.380 | 0.431 | 0.577 | 0.626 | 0.356 | 0.360 | 0.375 | 1 |

**S2 Table*.* Spearman’s *rho* Correlations Between the Items of the DASS-21**

All the correlations are significant at *p* < 0.01
