# Supplementary material for: Are anxiety, depression, and stress distinguishable in Italian adolescents? an examination through the Depression Anxiety Stress Scales-21
Source: PLoS One. 2024 Feb 27;19(2):e0299229. doi: 10.1371/journal.pone.0299229 (PMC10898757; doi:10.1371/journal.pone.0299229)
Supplement: S3 Table — (DOCX) [file pone.0299229.s003.docx]

**Are anxiety, depression, and stress distinguishable in Italian adolescents? An examination through the Depression Anxiety Stress Scales-21**

**Supporting information 3**

**S3 Table*.* Descriptive Indices of the DASS-21 in the Italian Adolescent Population (*N* = 655)**

|  | **Depression** | **Anxiety** | **Stress** | **Total** |
| --- | --- | --- | --- | --- |
| Mean | 13 | 8.44 | 13 | 25.6 |
| SD | 7.84 | 5.96 | 6.96 | 14.2 |
| 5th percentile | 2 | 1 | 2 | 5 |
| 10th percentile | 3 | 1 | 4 | 8 |
| 25th percentile | 6.5 | 4 | 8 | 14 |
| 50th percentile | 12 | 7 | 12 | 24 |
| 80th percentile | 20 | 14 | 20 | 38 |
| 85th percentile | 21.9 | 15 | 21 | 41 |
| 90th percentile | 24.6 | 17 | 23 | 45 |
| 95th percentile | 28 | 20 | 25 | 51 |

The mean scores were calculated based on the ESEM solution emerged in the present study, namely: Depression: items 3, 5, 7, 10, 12, 13, 14, 16, 17, 19, 21; Anxiety; items: 2, 4, 7, 9, 15, 17, 19, 20; Stress: items 1, 4, 6, 8, 11, 12, 14, 15, 16 18; Total: all items.
